# Modeling of Auditory Neuron Response Thresholds with Cochlear Implants

**DOI:** 10.1155/2015/394687

**Published:** 2015-07-05

**Authors:** Frederic Venail, Thibault Mura, Mohamed Akkari, Caroline Mathiolon, Sophie Menjot de Champfleur, Jean Pierre Piron, Marielle Sicard, Françoise Sterkers-Artieres, Michel Mondain, Alain Uziel

**Affiliations:** ^1^ENT Department and University Montpellier 1, University Hospital Gui de Chauliac, 34295 Montpellier, France; ^2^Audiology Department I-PAudioM, INSERM U1051 Unit, Institute for Neurosciences of Montpellier, 34081 Montpellier, France; ^3^Department of Medical Information and Biostatistics, University Hospital of Montpellier, Montpellier, France; ^4^U 1061, CIC 1001, INSERM, 34295 Montpellier, France; ^5^Neuroradiology Department, University Hospital Gui de Chauliac, 34295 Montpellier, France; ^6^Audiophonology and Speech Disorders Department, Institute Saint Pierre, 34250 Palavas, France

## Abstract

The quality of the prosthetic-neural interface is a critical point for cochlear implant efficiency. It depends not only on technical and anatomical factors such as electrode position into the cochlea (depth and scalar placement), electrode impedance, and distance between the electrode and the stimulated auditory neurons, but also on the number of functional auditory neurons. The efficiency of electrical stimulation can be assessed by the measurement of e-CAP in cochlear implant users. In the present study, we modeled the activation of auditory neurons in cochlear implant recipients (nucleus device). The electrical response, measured using auto-NRT (neural responses telemetry) algorithm, has been analyzed using multivariate regression with cubic splines in order to take into account the variations of insertion depth of electrodes amongst subjects as well as the other technical and anatomical factors listed above. NRT thresholds depend on the electrode squared impedance (*β* = −0.11 ± 0.02, *P* < 0.01), the scalar placement of the electrodes (*β* = −8.50 ± 1.97, *P* < 0.01), and the depth of insertion calculated as the characteristic frequency of auditory neurons (CNF). Distribution of NRT residues according to CNF could provide a proxy of auditory neurons functioning in implanted cochleas.

## 1. Introduction

Cochlear implants (CIs) allow the restoration of auditory perception through the direct electrical stimulation of the primary auditory neurons, located within the spiral ganglion of the cochlea. Typically, the outcome of CIs is assessed by evaluating the improvement in speech perception after implantation. However, speech perception is a high level function depending not only on CI functioning, but also on additional factors such as educational and speech therapy support after implantation, as well as the duration and cause of hearing loss, age, and educational level prior to it [1–3]. Thus, limiting the assessment of CI performance strictly to speech perception improvement does not properly evaluate the characteristics of the prosthesis-neural interface.

To study such an interface, electrophysiological testing should provide a more accurate proxy of the interaction between the electrodes of the CI and the auditory neurons. Among the different methods of measurements, electrically evoked compound action potentials (e-CAP) recorded within the CI may reflect this interaction. Indeed, the electrical current required to elicit an e-CAP may be directly correlated to neuronal density and excitability [4].

The use of e-CAP threshold recordings for CI fitting raised the interest of clinicians in the past 2 decades [5–7]. While correlations between psychophysical percepts and e-CAP thresholds with cochlear implant remain controversial, most authors agree that the “shape” of e-CAP thresholds represents a function of electrode position and follows the same distribution than psychophysical thresholds and comfort levels (T- and C-levels, [5, 6]).

Therefore, measuring e-CAP thresholds may provide a more accurate evaluation of residual auditory neuron population than speech perception testing. However, while literature is sparse regarding the impact of additional cues on e-CAPs, it is reasonable to assume that unknown factors could influence e-CAP thresholds. Indeed, if we refer to general principles of other electrically stimulating devices, electrode impedance and the distance between the electrode and the neurons should affect e-CAP thresholds. Other cochlear specific factors like the size of the cochlea, the depth of insertion, scalar placement of the electrodes, and obviously the number of residual functional auditory neurons, eventually associated with age and the duration of profound hearing loss, may also influence e-CAP thresholds [8–13].

In the present study, we propose a statistical model to describe the interactions between the cochlear neural response elicited by electrical pulses and biophysical, clinical, as well as cochlear specific factors in cochlear implanted subjects.

## 2. Methods

### 2.1. Population Study

This study, conducted under IRB approval, included data from 536 active electrodes in 31 subjects implanted between 2006 and 2012 (14 males, 17 females, mean age 32.3 yrs ± 10.5, min 17 yrs, max 63 yrs). Hearing loss etiology was progressive sensorineural deafness in 11 cases, genetic deafness in 8 cases, autoimmune disease in 4 cases, ototoxic medication in 4 cases, otosclerosis in 3 cases, and viral meningitis in 1 case.

The age and duration of profound hearing loss were respectively 40.26 ± 23.20 yrs (min 0, max 80 yrs) and 5.30 ± 5.32 (min 1 yr, max 30 yrs). Mean age at cochlear implantation was 45.56 ± 23.35 (min 4 yrs, max 84 yrs).

The CIs used were Nucleus (Cochlear) devices: CI24 RE CA with contour advanced (perimodiolar curved electrode array, PMA) in 27 patients (462 electrodes, 86.2%); CI24 RE ST with straight electrode array (SA) in 2 patients (31 electrodes, 5.8%); and CI422 with slim straight electrode array (SSA) in the remaining 2 patients (43 electrodes, 8%).

Full insertion of the electrode array through a round window approach was performed in all cases. Patients requiring cochlear reimplantation were excluded in order to avoid any additional factors that could potentially alter the CAP threshold.

### 2.2. Electrophysiological Recordings

All recordings were performed at least 6 months following implantation to ensure the stabilization of impedance levels. Electrophysiological recordings were performed on each active electrode at the time of referral.

Impedance of the recording electrodes and CAP (neural response telemetry NRT) thresholds were recorded with Custom Sound 4.0 software (Cochlear). All subjects were stimulated using the advanced combinational encoder (ACE) strategy at stimulation rates ranging between 900 and 1200 Hz. The impedances (kOhms) were recorded in MP1+2 and NRT thresholds (current level, C.L.) determined by the auto-NRT function on each active electrode [14]. Biphasic pulses were used at 80 pps rate with pulse duration of 25 *μ*s per phase and with an interpulse gap of 8 *μ*s. The stimulation started at relatively low intensity (100 C.L.) to avoid overstimulation in awake subjects. The thresholds were confirmed by the Auto-NRT algorithm using an optimization loop with ascending and descending series of stimulation to refine the threshold assessments.

### 2.3. Imaging Study

**Figure 1:**
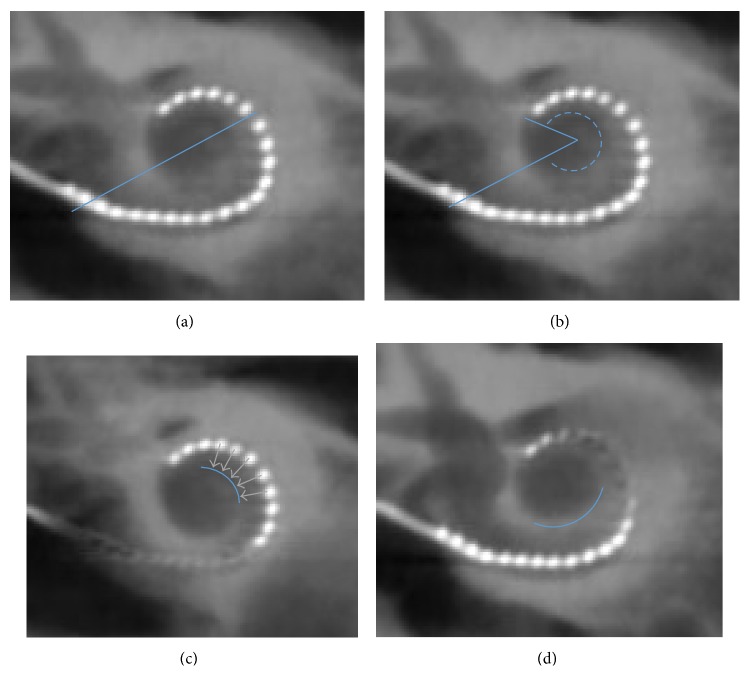
Calculation of the depth of insertion and distance between electrodes and the inner wall of the cochlea. (a) On an axial reconstruction, the large diameter of the cochlea was calculated to estimate the length of the cochlea as described by Escudé et al. (b) On an axial reconstruction, the angle of electrode array insertion was defined as that between the tangeant lines touching the most apical and the most basal electrodes. (c) On the same reconstruction, the distance between each electrode and the inner wall of the cochlea (outlined in blue) was calculated as the shortest orthogonal distance. (d) This calculation was repeated on several reconstructions to evaluate the whole electrode array.

**Figure 2:**
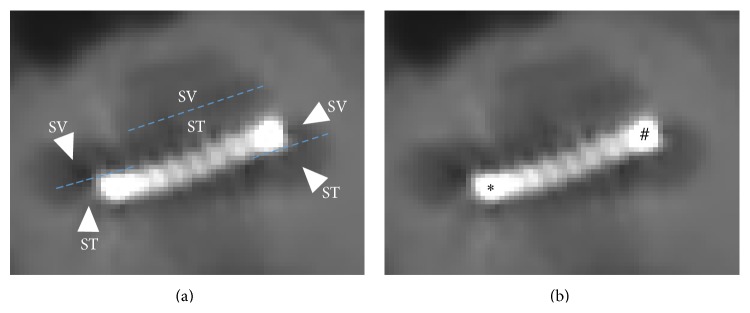
Midmodiolar reconstruction of cochlea implanted with contour advanced electrode array. (a) Several reconstructions were performed around the midmodiolar axis to evaluate the position of each electrode. After segmentation, each turn of the cochlea was separated into scala tympani (ST) or scala vestibuli (SV) compartments. (b) On this reconstruction we can see a scala vestibuli mislocation of the electrode array. Indeed the basal electrode (∗) is clearly located in the SV at the level of the first turn of the cochlea, whereas the apical one (**#**) is localized in the SV of the first turn of the cochlea.

Cone beam computed tomography was performed for every subject to evaluate the position of the electrode array in the cochlea (Newton 5G, 125 ∗ 125 ∗ 125 *μ*m voxel size for reconstructions). Axial and midmodiolar reconstructions were performed to evaluate the size of the cochlea, the angle of electrode array insertion, the distance between each electrode and the modiolus and the scalar placement of the electrodes. On the axial reconstruction, the large diameter of the cochlea was calculated to estimate the length of the cochlea as described by Escudé et al. [15] (Figure 1(a)). The depth of insertion was estimated using the angle of insertion from the round window to the most apical electrode (Figure 1(b)). The distance between each electrode and the modiolus was calculated as the shortest distance (perpendicular) between the center of the electrode and the inner wall of the cochlea (Figures 1(c) and 1(d)). Midmodiolar reconstructions enabled the localization of the scalar position for each electrode, that is, within the scala tympani (ST) or the scala vestibuli (SV) (Figures 2(a) and 2(b)).

The theoretical characteristic frequency of the neurons stimulated by each electrode contact was calculated using the Greenwood function modified by Stakhovskaya et al. [16], with the following parameters: cochlear duct length (CDL), insertion depth, electrode array length, and distance between electrode contacts for every electrode array subtype.

The CDL was calculated as described by Escudé et al. [15] by applying the following formula CDL = 2.62*A*∗ln⁡(1 + *θ*/235) = 4.3259*A*; with *A* equal to the length in mm of the large diameter of the cochlea (Figure 1(a)) and *θ* equal to the angle of a cochlea coiled on 2.75 turns (990°).

The relative position of each electrode was calculated as follows:

(1) $$\begin{array}{c} X_{e1}=\frac{\left(2.62A*\log\left(1+\theta_{e1}/235\right)\right)}{\text{CDL}},\\ X_{en}=\frac{\left[\left(2.62A*\log\left(1+\theta_{e1}/235\right)\right)-Y_{n}\right]}{\text{CDL}} \end{array}$$

*X*_*e*1_ = relative position of electrode 1 according to CDL, *X*_*en*_ = relative position of electrode *n* according to CDL, *θ*_*e*1_ = angle of insertion of electrode 1, *Y*_*n*_ = distance between electrode 1 and electrode *n* (in mm, according to manufacturer's data).

Then the cochlear place-frequency map for *n* was calculated according to Greenwood's function [17] for each electrode as follows:

(2) $$\begin{array}{c} {\text{CF}}_{\text{neur}(n)}=165.4*\left(10^{2.1Z_{en}}-0.88\right), \end{array}$$

with CF_neur(*n*)_ = characteristic neuron frequency at the position of the electrode *n*, *Z*_*en*_ = relative position of the neurons connected to the hair cells at the position *X*_*en*_.

*Z*_*en*_ was calculated using Stakhovskaya's formula [16].

(3) $$\begin{array}{c} Z_{en}=\frac{100}{\left[1+{\left(23/x_{en}-x_{en}/0.0099+0.76\right)}^{2}\right]}. \end{array}$$

### 2.4. Statistical Analysis

The characteristics of patients included in the present study are described with proportions for categorical variables and with mean and Standard Deviation (SD) values for continuous variables. When several measurements were made for a same subject, the mean of the average value for each subject was computed along with a within-subject SD (as described by Bland and Altman [18]) and between subject SD (on the average value of each subject). Interactions between NRT thresholds and neuron frequency per electrode were described graphically with mean and standard error of the mean (SEM).

Interactions between NRT threshold and demographical, clinical, and technical (of the CI) parameters were analyzed using a univariate linear mixed model to account for correlation between the repeated measurements of each subject (measurements of 22 different electrodes in the same subject, causing within-subject SD). For quantitative parameters, different types of relations were tested: linear, quadratic, cubic and polynomial. The type of association that maximized the Bayesian Index Criteria (BIC) was chosen to analyze the NRT threshold as a function of auditory neuron characteristic frequency. In order to model the complexity of the relation between NRT threshold and the characteristic neural frequency, we used a piecewise polynomial regression mixed model with cubic basis with two knots at 5000 and 10000 Hz using SAS PROC MIXED [19]. To analyze independent relations between NRT threshold and these parameters we used a multivariate linear mixed model, which included all the parameters. All the mixed models included a subject-specific random intercept. Significance of fixed effects was tested using the Wald Test. The relation between NRT threshold and the characteristic neural frequency is represented graphically with 95% confidence intervals. All statistical analyses were performed at the conventional two-tailed *α* level of 0.05 using the SAS statistical software (SAS Enterprise Guide 4.1, SAS Institute, Cary, N.C.).

## 3. Results

### 3.1. Analysis of Inter and Intra-Individual Variations of NRT Thresholds

**Figure 3:**
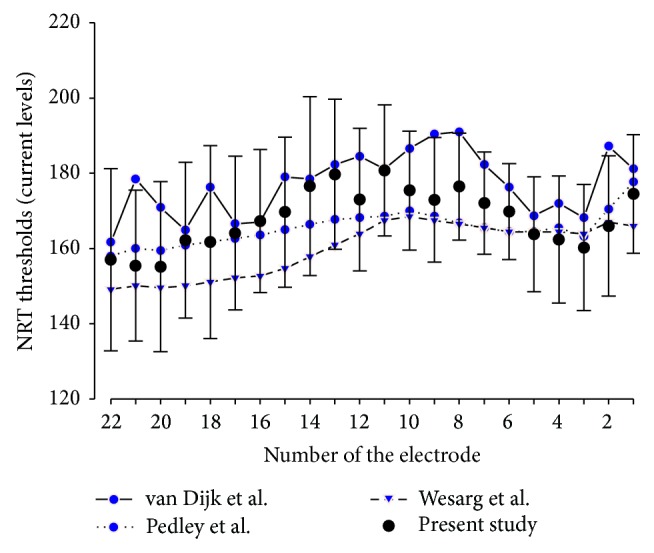
Mean NRT thresholds (±Standard Deviation) as a function of electrode number. Large interindividual variations of NRT threshold levels may be observed on the same electrode. No linear relationship is observed between NRT and electrode number as previously published.

In our population study, the mean impedance value was 8.71 kOhms (within subject SD 1.60, between subject SD 2.05, min 2.86, max 19.07 kOhms). Raw data from NRT thresholds spanned from 105 to 244 current levels (C.L., mean 169.68, within subject SD 13.66, between subject SD 18.60). The analysis of NRT thresholds electrode by electrode could not be summarized in a linear, quadratic, cubic, logarithmic or exponential relationship as shown in Figure 3.

We then evaluated the inter-individual variations of electrode arrays' insertion depth to determine if they could affect NRT thresholds. The average length of cochleae was 35.05 ± 4.68 mm (mean ± Standard Deviation consistently used throughout the text, min 26.38, max 43.42 mm). The mean depth of insertion of the electrode array was 343 ± 24 degrees (min 275, max 360°). According to the type of electrode array, mean insertion was 346 ± 22° with the PMA, 312 ± 46° with the SA, and 335 ± 35° with the SSA. All patients implanted with either an SA (CI24 ST) or an SSA (CI422) displayed the entire electrode array within the scala tympani, whereas 35.48% of patients with a PMA (CI24 CA) displayed a translocation of the electrode array within the scala vestibuli (Figure 2).

**Figure 4:**
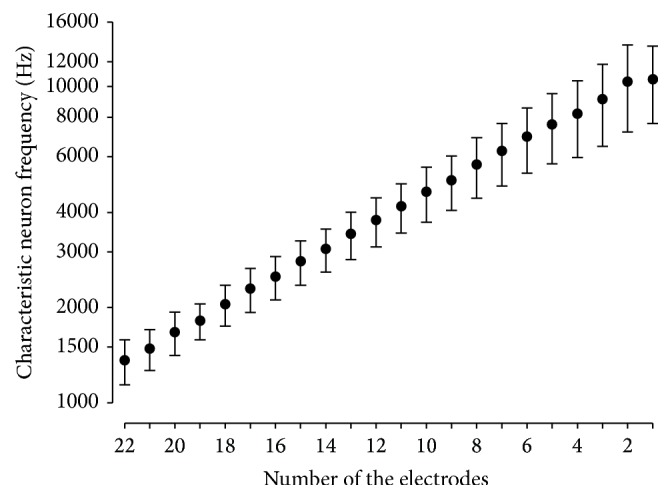
Mean characteristic auditory neuron frequency (±Standard Deviation) as a function of electrode number. Mean auditory frequency follows a linear relationship with electrode number, with a frequency increasing along with the number of the electrode. However, standard deviation bars show that the same frequency may be stimulated by up to 5 adjacent electrodes depending on the subject, thus arguing against the use of the electrode number to accurately evaluate insertion depth.

Considering that the observed inter-individual variations in depth of insertion may account for variations in NRT threshold, we plotted the NRT thresholds as a function of depth of insertion, calculated as the characteristic frequency of auditory neurons in the spiral ganglion (Figure 4). We transformed the variable depth of insertion (%) into characteristic frequency of auditory neurons (Hz) in order to use this variable for the modeling of the NRT thresholds using different types of regression models. Additionally, utilizing the report of characteristic frequency of auditory neurons instead of percentage of insertion depth allowed the use of a semi-logarithmic scale, which is more relevant for clinical application than relative depth of insertion.

**Figure 5:**
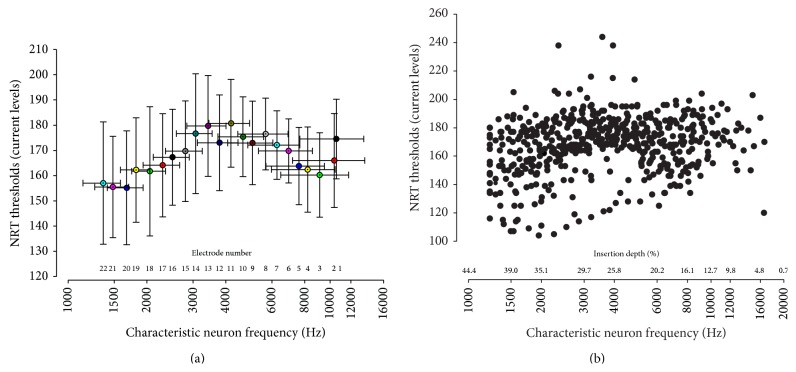
Mean NRT thresholds (±Standard Deviation) and mean characteristic auditory neuron frequency (±Standard Deviation) as a function of electrode number. Displaying NRT thresholds as a function of auditory neuron frequency for each electrode (a) clearly reveals the large variation of both NRT and frequency for the same electrode. Thus, a precise assessment requires the consideration of each electrode individually as a single point of coordinates (frequency, NRT), rather than the use of the electrode number (b).

As shown in Figure 4, the position of the electrode array within the cochlea may vary across subjects with a mean auditory neuron frequency ranging from a third to a full octave for the same electrode. Plotting NRT thresholds against characteristic neuron frequency for each electrode (Figure 5(a)) revealed the large variability of both NRT threshold and frequency for the same electrode, arguing against the use of electrode number as an independent variable. We therefore decided to use characteristic neuron frequency instead of electrode number in this study to provide a more accurate idea of electrode positioning within the cochlea (Figure 5(b)).

### 3.2. Statistical Analyses and Modeling of NRT Thresholds

First, a univariate linear mixed model was used to measure the effect of numerical variables (age at test, age of profound deafness, duration of profound deafness, age at implantation, impedance, distance electrode-lamina spiralis) and categorical variables (electrode position: ST/SV, type of electrode array: Slim Straight/Perimodiolar/Straight etiology of hearing loss: Progressive SNHL/Viral/Genetic/Autoimmune/Ototoxic/Otosclerosis) on NRT thresholds.

Univariate analysis showed a significant association of NRT thresholds with squared distance (*P* = 0.02) and a substantial although not significant association with squared impedance (*P* = 0.09). A significant (*P* = 0.04) negative association was found in subjects with ototoxic exposure. No association was found with age at evaluation, implantation or profound deafness, duration of the profound deafness, scalar position, and the type of electrode array (Table 1).

**Figure 6:**
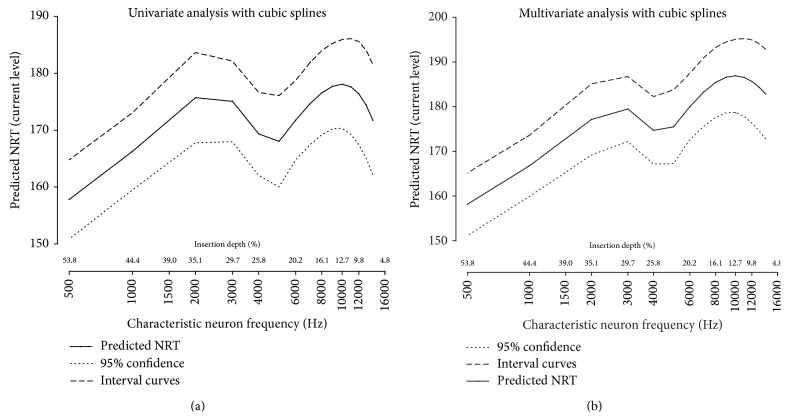
Modeling of NRT threshold as a function of characteristic auditory neuron frequency ((a): univariate regression, (b): multivariate regression including other variables). As can be seen, the prediction of NRT threshold level (black line) with 95% confidence interval (dashed lines), either univariate (a) or adjusted (b) for impedance and scalar placement (variables significantly associated with NRT threshold) shows an aspect of  “double bump” curve with a trend to thresholds elevation along with the increase of characteristic frequency of auditory neurons.

In order to model the complex relation between NRT threshold and the characteristic neural frequency, we used a piecewise polynomial regression mixed model with cubic basis with two knots at 5000 and 10000 Hz. Results of univariate regression are shown in Figure 6(a). After a steep increase of NRT between 500 and 3000 Hz, NRT thresholds dropped in the 4000–5000 Hz region before displaying a dome-like shape curve with a peak around 10000 Hz.

Using the same model, a multivariate analysis was performed to take into account the combined effects of numerical (including characteristic neural frequency) and categorical variables altogether. The multivariate model showed that NRT thresholds were negatively correlated (NRT threshold decreasing with an increasing variable value) with the scalar placement in the scala tympani (*P* < 0.01), and with the impedance of the electrodes (*P* < 0.01). This reveals that scala tympani placement of the electrode array led to a mean decrease of 8.50 ± 1.97 C.L. in NRT thresholds by comparison with scala vestibuli placement (Table 1). Every 1 kOhm elevation of electrode impedance resulted in a mean NRT threshold decrease of 0.11 ± 0.02 C.L. (Table 1).

In this multivariate analysis, no significant effect was found for the etiology of the hearing loss, the type of electrode array, the age at evaluation, implantation or profound deafness, or the duration of profound deafness. Although a close to significance, multivariate analysis did not support the significant difference found either for the ototoxic exposure (*P* = 0.06) in univariate analysis, or for the distance between each electrode and the inner wall of the cochlea (*P* = 0.13) (Table 1).

The relation between NRT thresholds and characteristic neuron frequency after adjustment on significant variables (squared impedance and scalar position) is represented in Figure 6(b). In addition to a similar “double bump” aspect of the univariate regression curve described above, a trend to an elevated threshold along with the characteristic neuron frequency was observed.

## 4. Discussion

Previous studies have failed in their attempts to correlate speech perception thresholds with most of the electrophysiological measurements performed with a CI [20, 21]. Indeed, speech perception not only relies on the efficacy of the electrical stimulation, but also involves central auditory pathways in addition to brain language areas and networks [1–3]. However, it is reasonable to assume that more specific psychoacoustical perceptions, like enhanced loudness growth or pitch perception [11, 12, 22] are directly linked to the activation of the residual auditory neurons by the electrical stimulation delivered by the electrode array. Indeed Cohen [12] and Kirby et al. [23] clearly showed that loudness growth function was proportional to e-CAP growth function, meaning that a stimulation with an increased current level causes an enhanced activation of auditory neurons, either directly by a recruitment of more neurons in the same region of the spiral ganglion, or indirectly by the spread of excitation of the electrical stimulation. However, e-CAP amplitude does not directly predict loudness, since it depends on additional factors discussed hereafter.

The relation between the electrodes and the neurons depends not only on the electrode array itself, but also on its insertion into the cochlea. Some authors recommend the use of perimodiolar arrays instead of straight arrays to favor a reduced NRT threshold. However, previous studies showed that perimodiolar placement of electrodes has no effect on NRT thresholds [24, 25], an observation consistent with our study.

On the other hand, perimodiolar arrays were associated with increased scala vestibuli mislocation in the present study and in several others [26, 27]. This type of mislocation does jeopardize the preservation of residual hearing and yet the use of perimodiolar arrays remains appealing in cases where no residual hearing needs to be preserved. Thus, it is also important to determine whether the scalar placement (scala tympani or vestibuli) alone impacts NRT thresholds. In the present study, we found a negative effect of scala vestibuli positioning of the electrodes on NRT thresholds. Therefore, in addition to favoring cochlear structure preservation, scala tympani insertion appears to be an important factor to reduce NRT thresholds. Furthermore, we suggest that preservation of such cochlear structure is also beneficial for speech perception performances with CI [2].

In this present study, the insertions of electrode arrays (perimodiolar and straight) were performed using a round window approach. However, the perimodiolar contour advanced arrays were initially designed to be inserted through a cochleostomy. The insertion of perimodiolar arrays through the round window is responsible of a deeper insertion and of a shortest distance between the basal electrodes and the modiolus [28], but may result in even more damages of cochlear structures [29, 30]. Thus the surgical approach, in addition to the electrode array subtype, has to be taken into consideration for NRT thresholds variability.

Increase in electrode impedance has been suggested to reflect the degree of cochlear fibrosis [31]. Indeed, changes in impedance in the postoperative period, followed by stabilization between 3 and 12 months postoperatively support this fact [32]. Fibrotic scars can reduce the performance of CIs by raising the threshold stimulation levels and energy consumption of the implant. Surprisingly, we found that electrodes with higher impedance had a moderate but significant reduction of NRT thresholds (average reduction of 0.11 C.L. per kOhm), regardless of the type of array or depth of insertion. Thus, it is conceivable that the fibrotic tissues surrounding the electrode array may contribute to focus the electrical current to the nearest wall of the cochlea and that the excitation does not spread into the spaces filled with perilymph. This reduction of NRT threshold could also be explained by a measurement artifact. Indeed, high impedance electrodes require more voltage to deliver the same current intensity. In this NRT recording paradigm, one cannot eliminate the effect of voltage in addition to current on either the generation of e-CAP measurements or on the recording by the automatic threshold detection algorithm. Similar variations of algorithm detection sensitivity have already been noticed by van Dijk et al. [14] and Wesarg et al. [33] when changing the pulse rate (80 versus 250 pps) and by consequences the charges per second.

Interestingly, we found a non-, but nearly, significant effect of the distance between the electrodes and the medial wall of the cochlea (where the spiral ganglion neurons are located) on NRT thresholds. The lack of statistical significance could reflect a limitation of our study, such as a lack of sensitivity of the CT cone beam reconstruction measurements to accurately evaluate this distance. In accordance with the role of electrode modiolus-distance, Holden et al. [2] found that a reduced distance between the electrodes and the modiolus, evaluated with cone beam imaging, could increase speech perception. Additional histological studies on implanted temporal bone specimens need to be conducted to further address this point.

Unlike speech perception results [1, 3], age at implantation, duration, and age of profound hearing loss had no significant effect on NRT thresholds. This observation supports the fact that these variables may act directly on the central auditory pathways and language areas with little or no effect on the peripheral auditory system.

In the present study, we found no impact of hearing loss etiology on NRT thresholds, despite a nearly significant effect of ototoxic exposure (drugs). This is consistent with the fact that aminoglycosides affect preferentially hair cells and stria vascularis and have minimal effects on auditory neuron survival (reviewed in [34]). Other deafness etiologies like otosclerosis, age-related hearing loss, genetic mutation, or noise may affect hair cells, the stria vascularis and neurons. Consequently, a more specific phenotyping would be necessary to identify specific cases in which auditory neurons are less damaged.

Only few studies have reported that speech perception outcomes may be related to etiology of hearing loss (reviewed in [3]). More particularly, some etiologies that affect cochlear anatomy, such as meningitis causing fibrosis, lead to poorer scores than Menière's disease, in which loss of neurons occurs later on [35]. This observation fits with our model, in which we studied separately the impact of etiology and the number of residual neurons through NRT thresholds. Our findings suggest that differences in speech perception outcome may be more connected to residual neurons than to the etiology of the loss of hearing itself.

In this study, we showed that NRT thresholds depend on the electrode depth of insertion into the cochlea.

While some studies report that NRT thresholds follow a base-apex gradient [36, 37], with basal electrodes near to the high frequency neurons displaying higher thresholds than apical electrodes placed closer to low-medium frequency neurons, others found no linear distribution of NRT thresholds [6, 14, 33, 38, 39] (see examples in Figure 3).

These differences could be explained by the number of electrodes used, instead of characteristic neuron frequency, resulting in an inaccurate localization of the electrode in the cochlea, in addition to a large variation of electrode positioning amongst subjects. As we have demonstrated, the depth of insertion of the electrode array, as well as the size of the cochlea, may vary between subjects and thus, estimation of the electrode depth of insertion using electrode number is insufficiently accurate.

In the present study, we noticed a “double bump” aspect of the relation between NRT thresholds and insertion depth, in addition to a moderate decrease of thresholds from the base to the apex of the cochlea. This report is to date, the first one using a multivariate analysis to describe separately the contribution of electrode insertion depth in addition to other factors on NRT thresholds values.

Assuming that the predicted NRT thresholds, calculated as a residual value of multivariate analysis, reflect indirectly the excitability level of the remaining auditory nerve fibers, one should expect that those values are a proxy of the functional auditory neuron population in the spiral ganglion neuron of implanted patients [4]. However, it is unclear why the obtained curve displays a “double bump” aspect with an elevation of thresholds at 10000 Hz (12.7% or 126° of cochlear length) and 3000 Hz (29.7% or 294° of cochlear length). Using perimodiolar arrays, Marx et al. [27] showed that a traumatism causing vestibular translocation of the electrode array may occur around 90–270° of insertion in 30% of cases. Thus, elevation of NRT thresholds may be caused by the traumatism of insertion, but does not explain the “double bump” ascending aspect of the curve, with a drop of NRT threshold at 4000 Hz.

Interestingly, the hearing sensitivity curve (ISO 226-2003 norm) of normal hearing individuals also displays a “double bump” ascending aspect of the curve by 500 Hz, with an increased sensitivity at 4000 Hz. Even if the hearing sensitivity of normal hearing individuals cannot be compared to the hearing sensitivity of implanted patients, it can be proposed that the increased sensitivity observed around 4000 Hz in normal hearing people relies, at least partially, on an increased capacity to recruit auditory neurons at this frequency, a hypothesis supported by our data in cochlear implant recipients.

Therefore, comparing the NRT threshold profiles of patients using our modeling might help in detecting subjects with abnormal neuron activation, and identify instances of auditory neuropathies or severe auditory neuron loss. This neuronal loss, in addition to personal or environmental factors (such as the duration of hearing loss, age of deafness, educational level, and speech therapy suppor) independent of cochlear implant characteristics, might explain cases of poor speech perception outcomes. Nevertheless, further histological studies will be needed to confirm this hypothesis.

## 5. Conclusion

With CIs, NRT thresholds vary according to the scalar placement of electrodes and their impedance. Scala tympani insertion is required not only to preserve residual hearing and cochlear structures, but also to improve the sensitivity of cochlear neurons.

Perimodiolar arrays did not display any advantage over straight arrays with regards to NRT thresholds and are associated with more frequent misplacement into the scala vestibuli. NRT thresholds, adjusted for scalar placement and impedance, may reflect auditory neuron sensitivity across the cochlea.

Ultimately, analyses of NRT thresholds might provide further information on the number of residual auditory neurons in each part of the cochlea and enable the evaluation of prosthetic-neural interface quality and consequential efficacy.

## Figures and Tables

**Table 1:** Parameter estimates of the linear mixed model evaluating the relation between neural response thresholds and demographical, clinical, and technical factors.

Fixed effect	Univariate analysis		Multivariate analysis	
	Crude *β*-estimate^*^ (SE)	*P* value^†^	Adjusted *β*-estimate^*^ (SE)	*P* value^†^
Age at test	0.13 (0.13)	0.31	0.29 (0.19)	0.11
Age of profound deafness	0.06 (0.12)	0.58	‡	—
Duration of profound deafness	−0.54 (0.48)	0.26	−0.17 (0.64)	0.78
Age at implantation	0.03 (0.11)	0.77	‡	—
Impedance ∗ impedance	**−0.03 (0.01)**	**0.09**	**−0.11 (0.02)**	**<0.01**
Distance ∗ distance	3.43 (1.44)	0.02	2.20 (1.46)	0.13
Neuron frequency	**Figure 6(a)**	**<0.01**	**Figure 6(b)**	**<0.01**
*Position *				
Scala vestibuli	**ref**		**ref**	
Scala tympani	**−0.21 (1.99)**	**0.91**	**−8.50 (1.97)**	**<0.01**
*Electrode array *				
Slim Straight	ref		ref	
Perimodiolar	−5.11 (12.95)	0.69	−5.94 (16.26)	0.71
Straight	1.02 (17.72)	17.72	13.43 (22.07)	0.54
*Etiology of hearing loss *				
Progressive SNHL	ref		ref	
Viral	0.53 (17.61)	0.97	2.55 (21.24)	0.90
Genetic	−5.36 (6.24)	0.39	−3.44 (7.22)	0.63
Autoimmune	−8.22 (9.74)	0.40	−10.50 (11.94)	0.37
Ototoxic	−19.34 (9.79)	0.04	−24.33 (13.24)	0.06
Otosclerosis	−2.23 (11.00)	0.83	−4.09 (13.45)	0.76

SE: standard error.

^*^ *β*-estimate: mean increase in NRT threshold according to the increase of one unit of the considered quantitative explanatory variable. For qualitative explanatory variables, the *β*-estimate is the mean difference in NRT compared to the reference category (ref).

^†^ *β*-estimates were compared to the value 0; a corresponding *P* value <0.05 indicates a significant association between NRT and the explanatory variable.

^‡^“Age of profound deafness” and “Age at implantation” were not entered into the multivariate model because of their high colinearity with “Age at test.”
